# Hearing and Neurological Impairment in Children with History of Exchange Transfusion for Neonatal Hyperbilirubinemia

**DOI:** 10.1155/2014/605828

**Published:** 2014-02-09

**Authors:** Carlos F. Martínez-Cruz, Patricia García Alonso-Themann, Adrián Poblano, Ileana A. Cedillo-Rodríguez

**Affiliations:** ^1^Department of Pediatric Follow-Up, National Institute of Perinatology, 11000 Mexico City, Mexico; ^2^Cognitive Neurophysiology Laboratory, National Institute of Rehabilitation, 14389 Mexico City, Mexico

## Abstract

The objective was to determine frequency of sensorineural hearing loss (SNHL), identified by abnormal threshold in evoked potentials, absence of otoacoustic emissions and behavioral responses, auditory neuropathy (AN) (absence of evoked potentials, with preservation of otoacoustic emissions), and neurological comorbidity in infants with hyperbilirubinemia (HB) treated with exchange-transfusion (ET). From a total of 7,219 infants, ET was performed on 336 (4.6%). Inclusion criteria were fulfilled in 102; 234 children did not meet criteria (182 outside of the study period, 34 did not have complete audiological evaluation, and 18 rejected the followup). Thirty-five children (34%) were born at-term and 67 (66%) were preterm. Children had a mean age of 5.5 ± 3.9 years. Main causes of ET were Rh isoimmunization in 48 (47%), ABO incompatibility in 28 (27.5%), and multifactorial causes in 26 (25.5%). Fifteen (15%) children presented with SNHL. Preterm newborns presented more often with SNHL. Indirect bilirubin level was higher in children with SNHL (22.2 versus 18.7 mg/dL, *P* = 0.02). No cases of AN were documented. An increased risk of neurologic sequelae was observed in children with SNHL. In conclusion, we disclosed a high frequency of SNHL in children with neonatal HB and ET and neurological alterations. No cases of AN were observed.

## 1. Introduction

The auditory pathway is known as one of the most susceptible parts of the central nervous system to noxious agents. Severe neonatal hyperbilirubinemia (HB) is a common cause of sensorineural hearing loss (SNHL) and auditory neuropathy (AN) [1–4]. If not controlled, HB can lead to hyperbilirubinemic encephalopathy, or neonatal death. Moreover, surviving infants are at high risk of neurological damage, which can manifest as cerebral palsy, epilepsy, SNHL, or cognitive deficits [5–9].

Some audiological studies in children with serum bilirubin levels >20 mg/dL, have reported auditory dysfunction in 17–87% of cases [10–14]. The usual treatments for this neonatal disease are phototherapy and blood exchange transfusion (ET). Phototherapy reduces serum bilirubin levels through luminous oxidation, while ET is used primarily to maintain bilirubin levels below toxicity levels, eliminate antibodies, and correct hemolytic anemia. However, some adverse events associated with ET are asymptomatic electrolyte and other blood abnormalities, which are treatable in the neonate. Overall, 74% of ET were associated with adverse events; the most common events were thrombocytopenia (44%), hypocalcemia (29%), and metabolic acidosis (24%), of which 69%, 74%, and 44%, respectively, required treatment [15, 16].

The Joint Committee on Infant Hearing of the American Academy of Pediatrics (AAP) considers ET a risk factor for SNHL [4]. SNHL, is a severe sensory sequelae in young infants and its early diagnosis depends on systematic hearing screening. Newborn hearing screening, mainly in high-risk infants, is the most effective way of early SNHL detection. Early diagnosis and intervention are crucial for improving linguistic development and prognosis of these children [4]. Therefore the main goal of this study was to determine the frequency of SNHL, AN, and neurological comorbidity in a group of children with a history of neonatal HB and ET treated at a third-level hospital in Mexico City.

## 2. Material and Methods

### 2.1. Subjects

We designed a retrospective, case-control study, with the following inclusion criteria: having been born at the National Institute of Perinatology “Dr. Isidro Espinosa de los Reyes” (INPerIER) in Mexico City, between January 1, 2000 and December 30, 2010; a history of ET secondary to severe HB after Rh hemolytic disease; ABO incompatibility or multifactorial HB, regardless of birth gestational age or associated morbidity during the neonatal period, and belonging to the pediatric followup clinic for high-risk newborns. Severe HB was defined as a bilirubin increase >0.5 mg/dL per hour in term infants, or >0.3 mg/dL for preterm infants, requiring exchange transfusion. Rh hemolytic disease was defined as different maternal-infant antigens and a positive direct Coomb's test. ABO incompatibility was defined as an infant's blood type A or B with a type O mother. Multifactorial HB was defined as the same maternal-infant blood type and severe HB. Our clinic's characteristics have been described in previous publications [17].

Phototherapy and ET were performed according to AAP guidelines [18–21]: (1) total serum bilirubin level over threshold values for ET according to gestational age and (2) increase of bilirubin >0.5 mg/dL per hour in term infants and >0.3 mg/dL for preterm infants regardless of phototherapy. ET was performed with a double transfusion volume (160 mL/kg) for term infants and (180 mL/kg) for preterm infants using compatible reconstituted fresh whole blood units.

Two groups were defined in the followup based on their hearing status: (1) children with SNHL and (2) a control group of children, consisting of eighty-seven children (85%), who showed bilateral normal hearing (BNH) with history of exchange transfusion for severe hyperbilirubinemia. Neonatal variables and procedures were compared as follows: gestational age at birth in weeks belongs to a term (birth age between 37 and 42 weeks) or preterm (<37 weeks) infant group; birth weight, Apgar score at one and five minutes, gender, days of endotracheal ventilation, length of hospital stay, and age at time of studies. Risk variables for SNHL documented in the neonatal period were peak serum indirect bilirubin level in mg/dL at the time of ET; days of phototherapy; exposure to other potentially ototoxic drugs such as aminoglycosides [22] and diuretics [23]; severe perinatal asphyxia (Apgar < 3 at one minute, pH < 7.25, PaO_2_ < 50 mmHg) [24]; intraventricular hemorrhage (determined by transfontanelar ultrasonography) during their stay in the neonatal intensive care unit (NICU) classified according to Papile et al. stages [25]. Exclusion criteria were as follows: family history of hearing loss; maternal/fetal infections in the first trimester of pregnancy (toxoplasmosis, rubella, cytomegalovirus, herpes virus, syphilis, human immunodeficiency); and congenital or metabolic diseases associated with hyperbilirubinemia such as: hereditary spherocytosis, thalassemia, Gilbert's syndrome, Crigler-Najjar disease, galactosemia, and glucose-6-phosphate dehydrogenase deficiency (diagnosis of these entities was carried out with the support of the genetics and hematology services of the hospital). Parents were informed of the importance of their child's participation, the purpose of the study, and research benefits. Causes for not participating in the pediatric followup were as follows: low economic resources, living far away from the hospital, and both parents working, among others reasons. Signed informed consent was requested when infants were recruited for the follow-up study, before hearing examinations in accordance with the institute's research committee and of the Declaration of Helsinki.

### 2.2. Bilirubin Determinations

Samples were obtained by peripheral venipuncture. All specimens were protected from light after they were drawn, and these were analyzed immediately. For the quantitative determination of serum bilirubin, we utilized a dichlorophenyl diazonium (DPD) reagent. Measurements were performed using a Beckman Synchron CX-9 equipment (Fullerton, CA, USA).

### 2.3. Brainstem Auditory Evoked Potentials (BAEP)

All infants included in the study underwent determination of conventional brainstem auditory evoked potentials (BAEP) at 3 and 6 months of chronological age with a Nicolet Viking Quest (Nicolet Biomedical Inc., Madison, WI, USA) computer. The test was conducted in a soundproof room reserved for this purpose within the neurophysiology unit, with the child in physiological sleep in a regular bed. BAEP determinations were performed after skin cleaning with alcohol-acetone and to apply conductive gel, using the international 10–20 system electrode placement [26] with the following assembly A1-Cz, A2-Cz. The studied ear was (−), Cz (+), and the contralateral ear was the ground. The electrode impedance was kept <4 Kilo-ohms. The band-pass filters were placed between 300 and 3,000 Hertz. The time of analysis after the stimulation was 10 milliseconds. Stimulation was carried out with monaural clicks in rarefaction at an intensity of 80 decibels (dB) of normal hearing level (nHL). The contralateral ear was simultaneously masked with a white noise 40 dB below the intensity of the stimulus. One thousand and five hundred clicks where administered for each sweep, decreasing in 20 dB steps to search for the threshold level in each ear. The duration of the stimulus was 100 microseconds and the clicks were delivered through TDH-49P headphones (Telephonics Co., Huntington, NY, USA). A normal peripheral auditory sensitivity was considered when the infant had a response to 40 dB nHL, displaying a robust positive wave V.

### 2.4. Tympanometry

To rule out middle ear pathology, children were studied with a Carl Zeiss OP-MI-9 F-125 Oto-microscope (Jenna, Germany). Afterward, we used a Grason-Stadler GSI TympStar V.2 Impedanciometer (Madison, WI), with ANSI S3.6-1996 calibration. The test tone used in tympanometry was 226 Hz, 85 at dB, pressure range = −600 to 400 deca-Pascals, compliance range of 0.1 to 5.0 mL, with an accuracy of ±5%. Children should have had a Jerger type A curve [27], with pressure variation of −150 to 50 deca-Pascals (to ensure a proper audiological test of quantitative and normative function in the assessment of middle ear and Eustachian tube).

### 2.5. Audiometry

Children >3 years of age underwent audiometry by conditioning game technique [28]. At 3 years the child must be able to react voluntarily to sounds, if given sufficient motivation. Once the child accepted the placement of TDH-50P balanced headphones, he was conditioned to put a toy in a rack, inserting it only during the test tone stimulus; this is repeated decreasing by 10 dB steps each time until the child no longer hears the test sound. After this, the test tone was increased by steps of 5 dB until it is perceived again, thus determining hearing thresholds for frequencies between 125 and 8,000 Hz in octave steps for each ear. The decrement-increment approach is the most commonly used technique in clinical audiology for determining hearing thresholds. We used a modified Hughson-Westlake method for children from sound to silence in steps of 10 by 10 dB and silence to sound in steps of 5 by 5 dB. We used a two-channel Grason-Stadler GSI 61 clinical audiometer with ANSI S3.43-1992 ISO 389 calibration and a bone vibrator placed on the forehead. For the contralateral ear, auditory masking white noise was used with automatic synchronization 10 dB below the level of the analyzed frequency. Audiometry was performed in a 3 m^2^ soundproof room.

Hearing was considered normal in conditioned audiometry when the threshold was ≤20 dB in the frequencies analyzed. The criteria for SNHL were considered when both the air and bone conduction thresholds were increased and overlapping with hearing thresholds ≥25 dB in at least two of the frequencies tested. All subjects were studied, diagnosed, and followed up by a certified pediatric audiologist (MCCF).

### 2.6. Evoked Otoacoustic Emissions

In order to document auditory neuropathy, automatic transient-evoked otoacoustic emissions (TEOAE) were performed in infants with abnormal BAEP result in both determinations. A Madsen otoacoustic-emission-analyzer AccuScreen GN Otometrics equipment (Copenhagen, Denmark) was utilized with the following technique: the study was conducted in a soundproof room, placing the probe in each of the ear canals. Stimulation was performed using clicks for each ear sweep. Equipment displays automatically a “Pass” or “Refer” result. “Pass” is equivalent to normal function of outer hair cells of the cochlea in the explored ear. The criteria for diagnosing AN consisted of two abnormal BAEP determinations (flat line or only wave V at high intensity stimulation) and “Pass” otoacoustic emissions result [2, 3].

### 2.7. Hearing Assessment Classification

Normal binaural hearing was considered when the infant passed the first or second test of conventional BAEP study, or when the infant passed the evaluation in the audiology clinic; these children formed the control group. SNHL was identified when the infant presented two BEAP studies with thresholds >45 dB nHL and did not pass the behavioral auditory tests. Hearing loss was classified in severity stages by averaging the hearing thresholds at 500, 1,000, and 2,000 Hz frequencies after performing the audiometric measurement for each ear. Subjects with audiometric threshold between 21 and 40 dB were classified with mild hearing loss; those between 41 and 70 dB with moderate hearing loss; children with thresholds of 71–90 dB were classified with severe hearing loss and >90 dB profound hearing loss [29].

### 2.8. Neurological Comorbidity

The presence of neurological sequelae (pathologic condition resulting from a disease, once the offending agent is removed) was documented by serial neurological examinations performed by a certified neuropediatrician with the help of brain imaging scans, neurophysiological recordings, laboratory studies, and with posterior appointments to the follow-up clinic to determine alterations such as cerebral palsy and/or epilepsy (according to International Classification of Diseases Tenth Edition, categories G80, and G40 resp.).

### 2.9. Statistical Analysis

Continuous data were presented as means and standard deviations and were analyzed using one-way analysis of variance (ANOVA) and the Mann-Whitney *U* test. Categorical variables are presented as percentages and were analyzed using the *χ* ^2^ test. Odds ratios (OR) were calculated for categorical variables for SNHL risk, with a statistical significance level of *P* < 0.05. For the data analysis we used the SPSS 17.0 for Windows (SPSS, Chicago, IL) [30].

## 3. Results

From a population of 7,219 children in the pediatric follow-up clinic for high-risk newborns, 336 (4.6%) children had undergone ET. One-hundred-two infants met inclusion criteria for this study, with a mean age of 5.5 years ±3.9 (range of 2 to 10 years), 234 children did not meet the inclusion criteria (182 were outside the study period, 34 did not have complete audiological evaluations, and 18 rejected the followup in the clinic). Causes of ET were distributed as follows: Rh isoimmunization, *n* = 48 (47%); ABO incompatibility, *n* = 28 (27.5%); and multifactorial HB, *n* = 26 (25.5%); the high number is possibly because our hospital is a referral center for high-risk pregnancies. Comparison of the mean values of indirect bilirubin and the frequency of SNHL among these three groups showed no differences (Table 1). Thirty-five children (34%) were born at term and 67 (66%) were preterm; we found fifteen patients (15%) with SNHL in our sample. We constructed a group of children with bilateral normal hearing (BNH) with 87 patients (85%) for comparison purposes.

Clinical characteristics of children with SNHL and BNH with ET are presented in Table 2. The mean Apgar score at one and five minutes was significantly lower for the group with SNHL. Children with SNHL had a lower gestational age at birth than children with BNH. Indirect serum bilirubin levels were significantly higher in the group of children with SNHL. Risk factors associated with neurological damage are presented in Table 3. Preterm birth, intraventricular hemorrhage, and exposure to furosemide were associated with SNHL.

BAEP results in children with SNHL were as follows: three infants had hearing thresholds of 80 dB nHL, two presented only wave V at 95 dB nHL, and ten had no response to >95 dB nHL stimulation. TEOAE recordings were negative in all cases of SNHL and thus, AN was not documented in the sample. Audiometric measurements showed severe SNHL in 10 cases (hearing threshold of 82 dB) with profound SNHL in 3 cases (hearing threshold of 99 dB). In all cases the auditory alteration was bilateral and symmetrical.

The higher neurological comorbidity was observed in the group of children with SNHL. An increased frequency of cerebral palsy was documented for the group with SNHL (20%) when compared with results from those of children with BNH (3%) (OR = 7.0 [1.2–38.7], *P* = 0.01). Epilepsy also showed a significant increased frequency in the group of children with SNHL (20%), when compared to children with BNH (5%) (OR = 5.1 [1.0–26.0], *P* = 0.02).

## 4. Discussion

### 4.1. Main Findings

This paper demonstrated a higher frequency of SNHL (15%) in children with a history of ET treated in a 3rd level hospital in Mexico City. Hearing alteration was produced despite the cause of severe hyperbilirubinemia and was associated to preterm birth and low gestational age, level of indirect bilirubin, and exposure to furosemide.

### 4.2. Comparison with Other Studies

Comparison with other studies is limited because of the differences in methodology, severity of hyperbilirubinemia, ET criteria, and SNHL classification. The basic mechanism of bilirubin neurotoxicity remains unknown. It is unclear why some infants do not develop hearing loss or neurological injury with the serum bilirubin levels that other infants do [9].

#### 4.2.1. Hearing Damage

Some researchers studied the effect of HB on the auditory pathway during its acute phase, with a short prospective design, assessing BAEP and otoacoustic emissions before and after phototherapy or ET [31, 32]; they found an increased risk for auditory damage in children with severe HB. Other studies have included only term infants with nonhemolytic jaundice, eliminating several risk factors and HB that cause hearing damage [33–36]. Some researchers have included the measurement of demographic or ethnic factors in their statistical analysis to weigh a multidimensional overview of the hearing damage after HB [37–39]. However, overall, their results usually coincide with our data, showing a higher frequency of auditory pathway dysfunction in children with severe HB and reports of alterations ranging from 17 to 87% of hearing dysfunction.

In this paper we analyzed the variable ET under usual clinical conditions present in the NICU, where newborns with severe HB usually present other associated co-morbidities, therefore being difficult to document severe HB as single disease. For example, Patra et al. [15] documented in a group of infants with history of neonatal ET that 62% also had other neonatal morbidities at the time of ET. In our sample, 50% of our children had other neonatal problems at the time of ET; thus, their results are in line with our observation.

Severe HB that requires ET for its treatment is a clinical variable that cannot be accurate or measured objectively, since it is not easy to precisely define a serum bilirubin value that indicates the need for ET or that is directly associated with neural or auditory damage. Thus, ET is a strong qualitative variable associated as a risk factor to SNHL. This paper demonstrates the need to pay special attention to the increased risk of SNHL among infants treated with ET for severe HB. However, not all cases of severe neonatal HB with ET result in hearing or neurological deficits, and the exact threshold in which bilirubin becomes dangerous is not uniform among populations.

HB is more prevalent and severe in preterm infants, and its course is more prolonged than in term infants as a result of the red blood cells, liver, and gastrointestinal system immaturity. As consequence of these facts, in this study we found an increased risk for SNHL in preterm infants.

Loop diuretics cause SNHL by inhibiting ion transport of within the stria vascularis, reducing the electrochemical gradients that create the Endocochlear potential. More important is the fact that loop diuretics enhance the rate of permanent hearing loss induced by aminoglycosides. The mechanism for interaction between aminoglycosides and loop diuretics implies alterations in the blood labyrinth barrier, which facilitates aminoglycoside entry to the endolymphatic compartment [23]. We do not know if this auditory lesion may include other ototoxic agents like indirect bilirubin. In this study we found that exposure to furosemide was associated with SNHL. Given the previous findings we suggest avoiding the use of aminoglycosides and furosemide combination in neonates undergoing ET to minimize the risk of SNHL.

#### 4.2.2. Auditory Neuropathy

The incidence of AN in infants with severe HB and ET has been reported as high [2, 12]. Nonetheless, our study did not document cases of AN, which could be due to the small number of children studied or to regional or ethnic considerations of the sample. Thus, the finding warrants future research to ascertain the nature of these differences.

The pathophysiology of SNHL secondary to severe HB is not well defined, although its toxicity can affect cochlear hair cells and neurons of the basal nuclei and of the central auditory pathways. A recent report of 30 infants with hearing loss and exposure to severe HB suggests that damage to the outer hair cells of the cochlea is very common; twenty-six infants (87%) out of 30 had cochlear damage and in four cases (13%) an AN was documented [40]. Audiological findings in the present study using BAEP, otoacoustic emissions and audiometry documented bilateral severe and profound SNHL with similar affection to both ears. Audiometry suggests damage to inner hair cells in the cochlea with greater injury to the basal turn (high tones) and manifestations of loss of sensitivity to sound stimuli. Abnormal results of otoacoustic emissions in our children with ET suggest also damage of the outer hair cells. This locates the auditory damage at the cochlear level and specifically at both: inner and outer hair cells of the organ of corti. As a secondary result, one of the main manifestations in neurodevelopment in these children may be a delay in language acquisition.

#### 4.2.3. Neurologic Comorbidity

Unfortunately the damage to the auditory pathway is not the only sequelae of severe HB. As we observed in our study, Ogunlesi et al. reported cerebral palsy in 86.4% of 22 infants with bilirubin encephalopathy, seizures in 40.9%, and deafness in 36.4% [8]. In our paper, we documented an increased risk of cerebral palsy and epilepsy for the SNHL group. Mechanisms for the alteration comprise the loss of neurons of the basal nuclei that result in motor disorders, death of cells, and formation of scars of the cerebral cortex manifested as seizures. Unfortunately, children with SNHL are frequently accompanied by other neurological or sensory deficits which may have a more difficult rehabilitation.

### 4.3. Study Limitations

The size of the sample was small, and therefore we must have caution in the interpretation of these results. In infants with HB treated with ET we reported here a higher frequency of SNHL and neurological comorbidity; however, we were unable to find AN. This fact merits more research in the future by our work team. These results deserve a continuous long-term pediatric followup of infants with greater number of patients.

## 5. Conclusions

The frequency of SNHL in children with a history of ET treated at a 3rd level hospital in Mexico City was high (15%). No cases of AN were documented. The preterm newborns have higher risk for SNHL. Moreover, children with SNHL and history of HB-ET have an increased risk of cerebral palsy and epilepsy. Thus, the early diagnosis and early intervention are very important actions for a better outcome of these patients.

## Figures and Tables

**Table 1:** Comparison of Indirect bilirubin levels in children with exchange transfusion for different causes (*n* = 102).

Causes of Exchange transfusion	SNHL			BNH			Both groups		
	* n *	IB mg/dL-sd	Range	* n *	IB mg/dL-sd	Range	* n *	IB mg/dL-sd	Range
Rh hemolytic disease	5	23.8 ± 7.2	16.2–35.6	43	18.0 ± 5.4	6.8–28.4	48	18.6 ± 5.8	6.8–35.6
ABO incompatibility	2	21.2 ± 9.2	14.7–27.8	26	18.7 ± 5.5	7.3–35.8	28	18.9 ± 5.6	7.3–35.8
Multifactorial hyperbilirubinemia	8	21.5 ± 4.5	16.1–29.3	18	20.2 ± 4.6	9.6–28.6	26	20.6 ± 4.5	9.6–29.3

Total	15	22.2 ± 5.7	14.7–35.6	87	18.7 ± 5.3	6.8–35.8	102	19.2 ± 5.4	6.8–35.8

*P* = one-way Anova		* P* = 0.77			*P* = 0.34			*P* = 0.31	

*n*: number of cases. Sd: standard deviation. IB: Indirect bilirubin. SNHL: sensorineural hearing loss. BNH: bilateral normal hearing. *P*: significance in one-way analysis of variance.

**Table 2:** Clinical characteristics of groups of children with SNHL and BNH with Exchange transfusion (*n* = 102).

Variable	SNHL group (*n* = 15)			BNH group (*n* = 87)				
	* n *	Average ± SD	Range	* n *	Average ± SD	Range	U M-W^a^	*P *
Gestational age (weeks)	15	33.7 ± 2.1	30–39.5	87	35.2 ± 3.3	26–40.6	425	0.03^a^
Birthweight (g)	15	1927 ± 581	910–2,975	87	2,218 ± 790	790–3795	514	0.19^a^
1 min Apgar score	15	6.4 ± 1.7	1–9	87	6.7 ± 2.2	2–9	497	0.12^a^
5 min Apgar score	15	8.3 ± 0.8	1–9	87	8.5 ± 1.0	4–9	504	0.08^a^
Mechanical ventilation (days)	5	5 ± 4	1–14	22	5 ± 2	1–20	46.5	0.58^a^
Hospital stay (days)	15	31 ± 26	7–96	87	20 ± 18	5–102	469	0.08^a^
Phototherapy (days)	15	5.5 ± 0.7	4–6	87	5.5 ± 1.4	1–10	637	0.87^a^
Indirect bilirubin (mg/dL)	15	22.2 ± 5.7	14.7–35.6	87	18.7 ± 5.3	6.8–35.8	451	0.05^a^
Age at follow-up (years)	15	6.7 ± 2.3	2–10	87	5 ± 3.7	1–10	566	0.41^a^
Male	9	60%		42	48%			0.40**
Female	6	40%		45	52%			
Term infant	1	7%		34	39%			0.01**
Premature infant	14	93%		53	61%			

SNHL: sensorineural hearing loss. BNH: bilateral normal hearing. *n*: number of cases. SD: standard deviation. ^a^ *U* M-W: *U* of Mann-Whitney test, **χ ^2^.

**Table 3:** Odds ratio calculations for risk-factors in children with SNHL and BNH with exchange transfusion.

Variable	SNHL *n* = 15		BNH *n* = 87		OR 95% CI	*P *
	Yes		Yes			
Preterm infants	14	93%	53	61%	8.9 (1.1–71.4)	0.01
Asphyxia	2	13%	5	6%	2.5 (0.4–14.3)	0.28
Neonatal sepsis	9	60%	42	48%	1.6 (0.5–4.9)	0.40
Intraventricular hemorrhage	2	13%	2	2%	6.5 (0.8–50.5)	0.04
Amikacin exposure	7	47%	49	56%	0.6 (0.2–2.0)	0.48
Furosemide exposure	5	33%	7	8%	5.7 (1.5–21.4)	0.005

SNHL: sensorineural hearing loss. BNH: bilateral normal hearing. *n*: number of cases. OR: odds ratio. CI 95%: confidence interval.
